# Real‐world KINDLE‐Latin America subset data on treatment patterns and clinical outcomes in patients with stage III non‐small‐cell lung cancer

**DOI:** 10.1002/cam4.4990

**Published:** 2022-07-04

**Authors:** Claudio Marcelo Martin, Adrián Puello‐Guerrero, Luis Alberto Mas‐Lopez, Saul Campos‐Gómez, Francisco J. Orlando‐Orlandi, Luis Fernando Tejado Gallegos, Reto Huggenberger

**Affiliations:** ^1^ Instituto Alexander Fleming Buenos Aires Argentina; ^2^ Universidad Autónoma de Santo Domingo (UASD), Instituto Nacional del Cáncer (INCART) Santo Domingo Dominican Republic; ^3^ Instituto Nacional de Enfermedades Neoplásicas and Oncosalud‐AUNA Lima Peru; ^4^ Centro Oncológico Estatal Instituto de Seguridad Social del Estado de México y Municipios Toluca Mexico; ^5^ Instituto Nacional del Tórax Santiago Chile; ^6^ AstraZeneca Mexico City Mexico; ^7^ AstraZeneca Baar Switzerland

**Keywords:** chemotherapy, lung cancer, NSCLC, radiotherapy

## Abstract

**Introduction:**

Stage III non‐small‐cell lung cancer (NSCLC) management is challenging given the heterogeneous nature of the disease. The LATAM subset of the real‐world, global KINDLE study reported the treatment patterns and clinical outcomes for LATAM from the pre‐immuno‐oncology era.

**Methods:**

The study was conducted in seven countries (Argentina, Chile, Colombia, Dominican Republic, Mexico, Peru and Uruguay) in stage III NSCLC (American Joint Committee on Cancer, 7th edition) diagnosed between January 2013 and December 2017. Retrospective data from patients' medical records (index date to the end of follow‐up) were collected. Summary statistics, Kaplan–Meier survival estimates and a two‐sided 95% confidence interval (CI) were provided. Cox proportional hazard model was used for univariate and multi‐variate analyses.

**Results:**

A total of 231 patients was enrolled, the median age was 65.0 years (range 21.0–89.0), 60.6% were males, 76.6% had smoking history, 64.0% had adenocarcinoma and 28.7% underwent curative resection. Multiple treatment regimens (>25) were used; chemotherapy alone was the most common (24.8%). The overall median progression‐free survival (mPFS) and median overall survival (mOS) were 14.8 months (95% CI, 12.1–18.6) and 48.6 months (95% CI, 34.7 to not calculable). Significantly better mPFS and mOS were observed for stage IIIA with curative surgery and resectable tumours and stage IIIB with an Eastern Cooperative Oncology Group score of 0/1, female gender, resectable tumours, adenocarcinoma and curative surgery (*p* < 0.05).

**Conclusion:**

Results show diversity in treatment practices and the corresponding clinical outcomes in stage III NSCLC. There is a need to streamline treatment selection and sequencing to decrease relapse rates after initial therapy.

## Graphical abstract

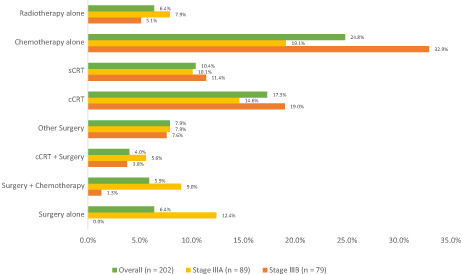

## 1 INTRODUCTION

Latin America (LATAM) is a diverse region with a population of about 600 million and more than 20 countries.^1^ In 2020, an estimated 97,601 new cases of lung cancer (6.6% of total cancer cases) and 86,627 lung cancer deaths were reported in LATAM and the Caribbean. Lung cancer was ranked first in cancer‐related deaths.^2^ The number of new lung cancer cases and deaths in women is expected to almost double in LATAM, whereas in men it is estimated to increase by 50% in LATAM and the Caribbean by 2030.^3^ Non‐small‐cell lung cancer (NSCLC) is the predominant subtype accounting for approximately 85% of all lung cancer cases.^4^ According to the American Joint Committee on Cancer (AJCC) staging system (7th edition), stage III includes two subtypes, IIIA and IIIB.^5^ In the recent AJCC edition (8th) published in 2017, stage IIIC was added to include locally advanced (LA) T3 and T4 tumours associated with N3 disease but without metastasis, reflecting their relatively worse prognosis compared with the prognosis of stage IIIB disease.^6^ About one‐third of all patients with NSCLC present with stage III LA disease.^7^

The heterogeneous nature of stage III disease makes the management challenging and often warrants an integrative multi‐modal treatment approach taking into account the demographic and clinical factors. Some stage III patients may benefit from surgery alone; however, for curative intent, (neo)adjuvant chemotherapy, concurrent chemoradiotherapy (cCRT) or sequential chemoradiotherapy (sCRT) is the standard of care (SoC). For patients with unresectable stage III disease, the SoC is platinum‐based doublet regimens comprising cisplatin/etoposide or carboplatin/paclitaxel combinations with concurrent radiotherapy and durvalumab as consolidation therapy in patients who have not progressed after ≥2 cycles of cCRT. The interim results of the ADAURA trial demonstrate that osimertinib has a definite role in treating resectable epidermal growth factor receptor (EGFR)‐mutated NSCLC after completion of curative‐intent surgery and adjuvant chemotherapy.^8^, ^9^, ^10^, ^11^, ^12^ The National Comprehensive Cancer Network (NCCN) 2021 guidelines now recommend adjuvant osimertinib for patients with completely resected EGFR mutation‐positive stage IIB‐IIIA NSCLC or high‐risk stage IB‐IIA NSCLC who received previous adjuvant chemotherapy or are ineligible to receive platinum‐based chemotherapy.^12^

More recently, IMpower010 showed a disease‐free survival benefit with atezolizumab versus best supportive care after adjuvant chemotherapy in patients with resected stage II–IIIA NSCLC, with pronounced benefit in the subgroup whose tumours expressed programmed death ligand 1 (PD‐L1) on 1% or more of tumour cells.^13^

The treatment practices in LATAM vary from country to country. A retrospective analysis of patient medical records (all stages of NSCLC) in a study in Uruguay showed chemotherapy or radiotherapy as first‐line treatment in 65.4% of cases^14^; whereas in Peru, patients with stage III NSCLC received chemoradiation (CRT) and occasionally surgery, where radiotherapy was used in the management of early, LA and metastatic lung cancer.^15^

Novel therapies including EGFR tyrosine kinase inhibitors (TKIs) have shown improvement in survival in patients with stage III NSCLC.^16^, ^17^, ^18^, ^19^, ^20^ However, patients in LATAM have not benefitted considerably from the advent of molecularly targeted therapies due to lack of uniformity in the availability of novel therapies across and within countries. Thus, the incidence/mortality ratio for lung cancer is considerably high in developing countries in LATAM than the developed countries.^1^ LATAM has a wide diversity in healthcare facilities, especially regarding the availability of specialised oncology care. Furthermore, there are limited existing databases or resources available that include information on diagnosis, treatment patterns and clinical outcomes of patients with stage III NSCLC. An understanding of real‐world treatment patterns of NSCLC is needed to evaluate whether the advent of new treatment modalities is translated into clinical practice in LATAM and its impact on clinical outcomes. A multi‐national real‐world KINDLE study was conducted to characterise treatment patterns and survival outcomes of patients with stage III NSCLC in Asia, the Middle East and Africa and LATAM.^21^ We report on treatment patterns and their associated clinical outcomes in patients with stage III NSCLC in the LATAM subset from the pre‐immuno‐oncology (pre‐IO) era. The study results have been reported in accordance with the Strengthening the Reporting of Observational Studies in Epidemiology checklist.^22^

## 2 METHODS

The LATAM subset of the non‐interventional, multi‐centre KINDLE study was conducted in seven countries (Argentina, Chile, Colombia, Dominican Republic, Mexico, Peru and Uruguay) across 13 centres (Supplementary Figure S1). Retrospective data were collected for patients with stage III NSCLC. The study was conducted in accordance with the Declaration of Helsinki, International Council for Harmonisation, Good Clinical Practice, Good Pharmacoepidemiology Practice and the applicable legislation for non‐interventional studies.

### 2.1 Study population

The study population included adult patients aged ≥18 years, diagnosed with de novo LA stage III NSCLC (AJCC 7th edition) between January 2013 and December 2017 and with medical records available for at least 9 months from the date of initial diagnosis (index date). Patients with concomitant cancer at the time of diagnosis of stage III NSCLC or within 5 years before NSCLC diagnosis, except for non‐metastatic, non‐melanoma skin cancers or in situ or benign neoplasms were excluded as were patients with an initial diagnosis of stage I to II NSCLC and diagnosed with stage III disease at the time of relapse. Patients who participated in clinical trials were not specifically excluded from this study.

### 2.2 Data collection

Extracted data from available medical records were entered in the electronic case report forms from the date of initial diagnosis to the end of the follow‐up (defined as death, last available medical record or end of data collection, whichever occurred first). Baseline data included demographic characteristics (age, gender and body mass index) and smoking status, clinical characteristics (Eastern Cooperative Oncology Group [ECOG] performance status, histology, EGFR mutation status, PD‐L1 status and stage as per AJCC 7th edition) and treatment patterns (treatment modality and line of treatment). The occurrence and date of disease progression were determined from patients' medical records such as imaging reports, pathology reports and oncologists' notes and statements on disease progression. Overall survival (OS) was defined as the time from stage III NSCLC diagnosis or time from treatment initiation until death from any cause. Real‐world progression‐free survival (PFS) was defined as the time from treatment initiation to documented disease progression or death due to any cause, whichever occurred first.

### 2.3 Statistical analyses

Summary statistics were used to describe patient demographics, clinical characteristics and treatment modalities. Categorical variables were summarised using frequencies and percentages and continuous variables by descriptive statistics including median, minimum and maximum, with a 95% confidence interval (CI). The treatment patterns and their associated clinical outcomes were analysed for the overall cohort followed by further analyses in patients with stage IIIA/IIIB and resectable/unresectable disease. Median survival estimates (OS and PFS) were determined descriptively using the Kaplan–Meier survival curves. The median survival estimates were reported along with the two‐sided 95% CI. A multi‐variate Cox proportional hazards model and hazards ratio along with 95% CI were used to identify the significant effects of clinical and demographic factors on OS and PFS by controlling relevant demographic and clinical covariates affecting OS and PFS. A *p* value of <0.05 was considered statistically significant. SAS9.4 was used to perform the analyses.

## 3 RESULTS

### 3.1 Sociodemographic and clinical characteristics

A total of 231 patients with stage III NSCLC from seven countries in the LATAM region participated in the KINDLE study. A total of 142 (61.5%) patients were alive at the time of data collection. A majority of patients were from Argentina (33.8%) followed by Chile (21.6%). The median (range) duration of follow‐up for patients was 660 days (7–2404). The median (range) age of patients was 65.0 years (21.0–89.0), most were men (60.6%) and more than three‐fourths (76.6%) were current or past smokers. A majority of patients were classified as per AJCC 7th edition staging criteria (stage IIIA: 103 [53.4%] and stage IIIB: 90 [46.6%]), with adenocarcinoma being the predominant (146 [64.0%]) histological type. Only 35 patients were classified as per AJCC 8th edition staging criteria (stage IIIA: 19; stage IIIB: 16; none with stage IIIC). Most patients had T3 (32.5%) or T4 (28.5%) tumours, with nodal involvement N2 (59.2%) and N3 (15.8%). Among patients with available ECOG performance status at diagnosis (*n* = 134), 119 (88.8%) had a performance score of ≤1. EGFR mutation testing was performed for about 30% of patients (74/231) of which 21 (28.4%) were positive, while PD‐L1 testing was performed for about 10% of patients (22/231) of which 14 (63.6%) were positive. At the time of primary diagnosis, molecular testing was performed for 38.2% and 41.6% of patients with stage IIIA and IIIB disease and 41.7% and 41.2% of patients with resectable and unresectable disease, respectively. A total of 43.0% of cases were discussed in the multi‐disciplinary team meeting. Table 1 provides the sociodemographic and clinical characteristics for the LATAM subset.

**TABLE 1:** Baseline sociodemographic and clinical characteristics of patients with stage III NSCLC in KINDLE LATAM. The frequency and distribution of the sociodemographic and clinical variables are depicted

Parameters	Latin America (*N* = 231)
Age (years), median (range)	65.0 (21–89)
Gender, male, *n* (%)	140 (60.6)
BMI (kg/m^2^), median (range)	24.8 (17–37)
Vital status, *n* (%)	
Alive	142 (61.5)
Dead	89 (38.5)
Smoking status^a^, *n* (%)	
Current smoker	83 (35.9)
Ex‐smoker	94 (40.7)
Never smoker	37 (16.0)
Current average cigarettes per day, median (range)	20 (1–80)
Number of pack years, median (range)	40 (1–180)
Ethnicity, *n* (%)	
Caucasian	69 (29.9%)
East Asian	1 (0.4%)
Hispanic	23 (10.0%)
Mestizo	58 (25.1%)
Mixed (e.g. brown, mulato, others)	11 (4.8%)
Other	1 (0.4%)
Unknown	68 (29.4%)
AJCC stage (7th edition), *n* (%)	
Stage IIIA	103 (53.4)
Stage IIIB	90 (46.6)
Histology type, *n* (%)	
Adenocarcinoma	146 (64.0)
Squamous cell/epidermoid carcinoma	54 (23.7)
Other	11 (4.8)
Large cell carcinoma	10 (4.4)
Mixed	2 (0.9)
ECOG performance status, *n* (%)	
0	65 (48.5)
1	54 (40.3)
≥2	15 (11.2)
T stage, *n* (%)	
T1	0
T1a	9 (3.9)
T1b	5 (2.2)
T1c	3 (1.3)
T2	0
T2a	34 (14.9)
T2b	22 (9.6)
T3	74 (32.5)
T4	65 (28.5)
TX	7 (3.1)
N/A	9 (3.9)
N stage, *n* (%)	
N0	15 (6.6)
N1	31 (13.6)
N2	135 (59.2)
N3	36 (15.8)
NX	11 (4.8)

*Note*: Unknown and missing data are not included.

Abbreviations: AJCC, American Joint Committee on Cancer; BMI, body mass index; ECOG, Eastern Cooperative Oncology Group; NSCLC, non‐small‐cell lung cancer; TNM, tumour, node, metastasis.

^a^ Pack‐years is the product of the number of years smoked and the average number of packs per day.

### 3.2 Treatment patterns

**FIGURE 1:**
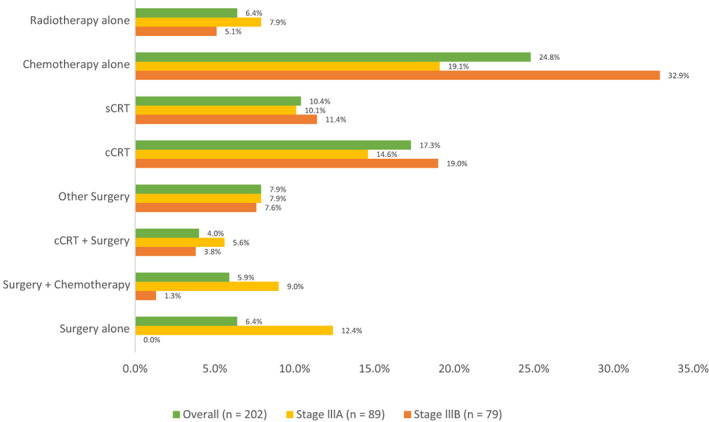
Frequent treatment modalities for stage III NSCLC initial Therapy. The frequency of the various initial treatment modalities used, are shown by stage (IIIA vs IIIB) and overall. cCRT, concurrent chemoradiotherapy; NSCLC, non‐small‑cell lung cancer; sCRT, sequential chemoradiotherapy. Note: The cut‑off used for selection of treatment modalities is 5% in either of the groups.

Of the 231 patients enrolled in the LATAM subset, 87.4% (202/231) received initial therapy (IIIA: 86.4% [89/103], IIIB: 87.8% [79/90]). Post‐relapse, 65 patients received a second‐line treatment and only 18 patients received a third‐line treatment. About 12% of patients (*n* = 29) did not receive any treatment for NSCLC. Figure 1 shows the most common treatment modalities administered under initial therapy. All treatment modalities are summarised in Supplementary Data Tables S1 and S2. Pooling the patients who underwent surgery (alone or with [neo]adjuvant) as part of initial therapy showed about one‐fourth of patients (58 [28.7%]) underwent surgical resection (IIIA: 34 [38.2%], IIIB: 13 [16.5%]); of these, 19 (32.8%) patients received adjuvant treatment. Lobectomy was the most frequent (46 [79.3%]) type of surgery. Of the 30 treatment modalities under initial therapy, top 3 modalities were chemotherapy alone (50 [24.8%]), cCRT (35 [17.3%]) and sCRT (21 [10.4%]). Treatment patterns were similar for patients with stage IIIA and IIIB NSCLC, with chemotherapy alone as the most frequent modality in both the groups (17 [19.1%] and 26 [32.9%]), followed by cCRT (13 [14.6%] and 15 [19.0%]) and sCRT (9 [10.1%] and 9 [11.4%]). In patients with resectable NSCLC, 84.7% (50/59) of patients underwent surgical resection (alone or with [neo]adjuvant), whereas for patients with unresectable disease, chemotherapy alone (32.2% [37/115]) was the most frequent modality.

### 3.3 Recurrence and survival outcomes

After initial therapy, 67.0% (136/203) of patients relapsed (had disease progression or died). Among patients with stage IIIA and IIIB NSCLC, 70.8% (63/89) and 73.8% (59/80) patients relapsed. After initial therapy, 49.2% (29/59) of patients with resectable NSCLC relapsed. In patients with unresectable disease, the relapse rate was higher (75.0% [87/116]). Among patients who received second‐line treatment, the most frequent modality was chemotherapy alone (23 [35.4%]), followed by targeted therapy (9 [13.8%]) and immunotherapy (6 [9.2%]). Of patients receiving any third‐line treatment, most received chemotherapy alone and targeted therapy (4 [22.2%] each) followed by radiotherapy alone, immunotherapy and chemotherapy + immunotherapy (2 [11.1%] each).

**FIGURE 2:**
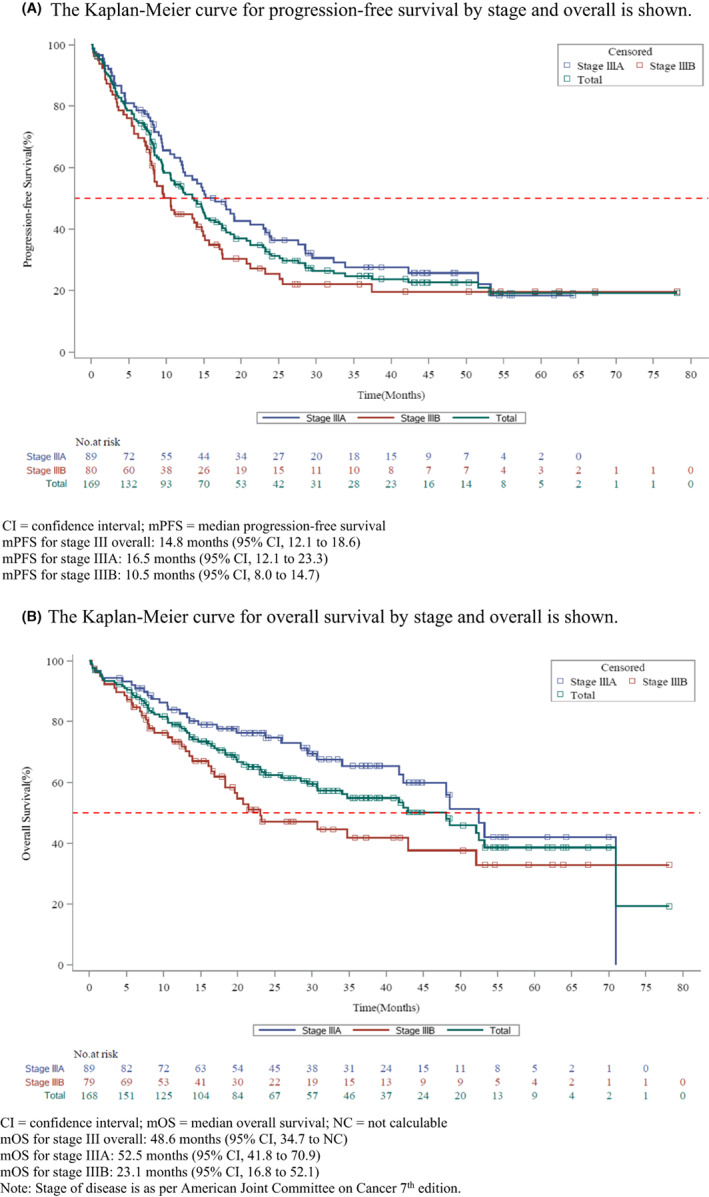
(A) Kaplan–Meier plot of progression‐free survival after initial therapy by disease stage. The Kaplan‐Meier curve for progression‐free survival by stage and overall is shown. CI, confidence interval; mPFS, median progression‐free survival. mPFS for stage III overall: 14.8 months (95% CI, 12.1–18.6); mPFS for stage IIIA: 16.5 months (95% CI, 12.1–23.3); mPFS for stage IIIB: 10.5 months (95% CI, 8.0–14.7). Note: Stage of disease is as per American Joint Committee on Cancer 7th edition. (B) Kaplan–Meier plot of overall survival after initial therapy by disease stage. The Kaplan–Meier curve for overall survival by stage and overall is shown. CI, confidence interval; mOS, median overall survival; NC, not calculable. mOS for stage III overall: 48.6 months (95% CI, 34.7 to NC); mOS for stage IIIA: 52.5 months (95% CI, 41.8–70.9); mOS for stage IIIB: 23.1 months (95% CI, 16.8–52.1). Note: Stage of disease is as per American Joint Committee on Cancer 7th edition.

The median PFS (mPFS) and median OS (mOS) after initial therapy was 14.8 months (95% CI, 12.1–18.6) and 48.6 months (95% CI, 34.7 to not calculable [NC]). The mPFS for stage IIIA was 16.5 months (95% CI, 12.1–23.3), and for stage IIIB, it was 10.5 months (95% CI, 8.0–14.7); the mOS for stage IIIA was 52.5 months (95% CI, 41.8–70.9), and for stage IIIB, it was 23.1 months (95% CI, 16.8–52.1; Figure 2A,B). The mPFS was 30.3 months (95% CI, 18.0–NC) for patients with resectable NSCLC and 11.1 months (95% CI, 8.3–14.7) for patients with unresectable disease. The mOS was NC in patients with resectable NSCLC and was 36.5 months (95% CI, 19.9–48.0) for patients with unresectable disease. Survival outcomes based on treatment modality received in patients with resectable NSCLC showed mPFS of surgery + adjuvant chemotherapy to be significantly longer (*p* < 0.05) than that of either radiotherapy or chemotherapy alone. However, there was no significant difference in mOS between these groups. In the unresectable category, mPFS for cCRT and sCRT were significantly longer (*p* < 0.05) than that for chemotherapy alone or radiotherapy alone. However, there were no significant differences in mOS of cCRT and sCRT and other treatment modalities.

Survival outcomes based on treatment modality received in patients with stage IIIA showed mPFS of 9.0 months (95% CI, 3.2–19.1) with the most frequent modality of chemotherapy alone. The mPFS was the longest in patients who received cCRT (21.2 months; 95% CI, 2.7–NC) followed by patients who received targeted therapy (20.3 months, 95% CI, 8.3–32.3). In stage IIIB, mPFS in patients who received the most frequent modality of chemotherapy alone was 7.3 months (95% CI, 3.2–9.5). The mPFS was the longest in patients who received sCRT (17.5 months; 95% CI, 6.2–NC) followed by patients who received surgery + chemotherapy alone (14.1 months; 95% CI, NC to NC). In stage IIIA, mOS in 17 patients who received the most frequent modality of chemotherapy alone was 25.9 months (95% CI, 9.5–53.2). Nine patients who received sCRT had the longest mOS (70.9 months; 95% CI, 0.7–70.9) followed by 13 patients who received cCRT (48.0 months; 95% CI, 5.7–NC). In stage IIIB, mOS in 26 patients who received the most frequent modality of chemotherapy alone was 18.3 months (95% CI, 13.0–NC). The mOS was the highest in two patients who received chemotherapy + targeted therapy at 36.8 months (95% CI, 30.7–42.9) (Supplementary Table S3 and S4).

### 3.4 Logistic regression analysis

The effect of various sociodemographic and clinical characteristics on PFS and OS using univariate and multi‐variate analyses by stage of NSCLC is shown in Table 2 (stage IIIA) and Table 3 (stage IIIB). Univariate and multi‐variate analyses for the overall cohort are presented in Supplementary Data Tables S5 and S6. For stage IIIA, univariate analyses showed significantly longer mPFS and mOS in patients with resectable tumours (hazard ratio [HR], 0.41 [95% CI, 0.23–0.72], *p* = 0.0018 and HR, 0.26 [95% CI, 0.11–0.62], *p* = 0.0023) and those who underwent surgery as part of initial therapy (HR, 0.42 [95% CI, 0.24–0.74), *p* = 0.0023 and HR, 0.37 [95% CI, 0.16–0.82), *p* = 0.0152). For stage IIIB, mPFS and mOS were significantly longer for patients with an ECOG performance status score of 0/1 (HR, 0.40 [95% CI, 0.17–0.93], *p* = 0.0325 and HR, 0.34 [95% CI, 0.14–0.86], *p* = 0.0229), female gender (HR, 2.28 [95% CI, 1.30–4.0], *p* = 0.0042 and HR, 2.11 [95% CI, 1.06–4.20], *p* = 0.0337), resectable tumours (HR, 0.34 [95% CI, 0.14–0.80], *p* = 0.0140 and HR, 0.24 [95% CI, 0.07–0.80], *p* = 0.0203), and who underwent surgery as part of initial therapy (HR, 0.33 [95% CI, 0.14–0.77), *p* = 0.0103 and HR, 0.16 [95% CI, 0.04–0.67], *p* = 0.0121). Patients with histology of adenocarcinoma had longer mOS compared to other histologies (HR, 0.40 [95% CI, 0.21–0.76], *p* = 0.005). In stage IIIB disease, non‐smokers had significantly better mPFS (HR, 3.02 [95% CI, 1.35–6.736], *p* = 0.0069), but there was no impact on the mOS.

**TABLE 2:** Univariate and multi‐variate analyses for survival outcomes for stage IIIA NSCLC based on sociodemographic characteristics and treatment regimen in KINDLE LATAM

	Stage IIIA					
	Univariate analyses mPFS			Multi‐variate analyses mPFS		
	Numbers	HR (95% CI)	*p* value	Numbers	HR (95% CI)	*p* value
Age > 65 vs ≤65	48 vs 41	0.584 (0.355 to 0.961)	0.0342	17 vs 23	0.233 (0.084 to 0.647)	0.0052
ECOG 0/1 vs 2/3/4	39 vs 5	0.675 (0.233 to 1.955)	0.4690	36 vs 4	0.310 (0.061 to 1.570)	0.1572
EGFRm vs EGFR WT	9 vs 18	0.463 (0.187 to 1.149)	0.0969	—	—	—
Male vs Female	50 vs 39	0.958 (0.583 to 1.575)	0.8664	24 vs 16	0.751 (0.269 to 2.092)	0.5833
Smoking history, yes vs no	70 vs 12	1.414 (0.666 to 3.002)	0.3669	32 vs 8	1.200 (0.389 to 3.698)	0.7507
Resectable, yes vs no	36 vs 41	0.410 (0.234 to 0.718)	0.0018			
Adenocarcinoma vs Others	59 vs 29	0.811 (0.482 to 1.365)	0.4301	26 vs 14	0.933 (0.346 to 2.519)	0.8913
Surgery in first‐line, yes vs no	34 vs 55	0.422 (0.242 to 0.735)	0.0023	16 vs 24	0.303 (0.104 to 0.886)	0.0291
cCRT alone in first‐line, yes vs no	13 vs 76	0.904 (0.430 to 1.900)	0.7893	6 vs 34	1.258 (0.359 to 4.404)	0.7194
cCRT alone in first‐line vs sCRT alone in first‐line	13 vs 9	0.887 (0.321 to 2.452)	0.8165	—	—	—
Trimodality in first‐line, yes vs no	7 vs 82	0.647 (0.235 to 1.784)	0.4003	4 vs 36	0.392 (0.043 to 3.589)	0.4072

	Stage IIIA					
	Univariate analyses mOS			Multi‐variate analyses mOS		
	Numbers	HR (95% CI)	*p* value	Numbers	HR (95% CI)	*p* value
Age > 65 vs ≤65	48 vs 41	0.966 (0.480 to 1.945)	0.9228	17 vs 23	0.479 (0.134 to 1.718)	0.2589
ECOG 0/1 vs 2/3/4	39 vs 5	0.734 (0.164 to 3.286)	0.6865	36 vs 4	0.906 (0.095 to 8.674)	0.9318
EGFRm vs EGFR WT	9 vs 18	0.151 (0.019 to 1.207)	0.0746	—	—	—
Male vs female	50 vs 39	1.537 (0.744 to 3.174)	0.2454	24 vs 16	1.050 (0.226 to 4.882)	0.9499
Smoking history, yes vs no	70 vs 12	1.122 (0.421 to 2.988)	0.8181	32 vs 8	1.305 (0.234 to 7.281)	0.7615
Resectable, yes vs no	36 vs 41	0.263 (0.111 to 0.621)	0.0023	—	—	—
Adenocarcinoma vs others	59 vs 29	0.510 (0.253 to 1.029)	0.0601	26 vs 14	1.024 (0.263 to 3.981)	0.9726
Surgery in first‐line, yes vs no	34 vs 55	0.366 (0.163 to 0.824)	0.0152	16 vs 24	0.124 (0.014 to 1.103)	0.0613
cCRT alone in first‐line, yes vs no	13 vs 76	1.896 (0.778 to 4.618)	0.1591	6 vs 34	2.734 (0.484 to 15.454)	0.2550
cCRT alone in first‐line vs sCRT alone first‐line	13 vs 9	2.294 (0.460 to 11.429)	0.3109	—	—	—
Trimodality in first‐line, yes vs no	7 vs 82	1.588 (0.554 to 4.549)	0.3892	4 vs 36	2.796 (0.135 to 57.864)	0.5059

*Note* 1: Stage of tumour is per AJCC 7th edition.

*Note* 2: In the univariate analysis, each time one variable is considered, only patients with missing data in that variable were excluded. While, in the multi‐variate analysis, patients with missing data in any of the variables input to the model were excluded. So the numbers for univariate and multi‐variate analyses differ. The variables for multi‐variate analysis are based on the univariate analysis results and also the assumptions for multi‐variate Cox proportional hazards model are valid.

Abbreviations: AJCC, American Joint Committee on Cancer; cCRT, concurrent chemoradiotherapy; CI, confidence interval; EGFR, epidermal growth factor receptor; ECOG, Eastern Cooperative Oncology Group; HR, hazard ratio; mOS, median overall survival; mPFS, median progression‐free survival; NC, non‐calculable; NSCLC, non‐small‐cell lung cancer; sCRT, sequential chemoradiotherapy; TNM, tumour, node, metastasis; WT, wild type. ‐ indicates not available.

**TABLE 3:** Univariate and multi‐variate analyses for survival outcomes for stage IIIB NSCLC based on sociodemographic characteristics and treatment regimen in KINDLE LATAM

	Stage IIIB					
	Univariate analysis mPFS			Multi‐variate analysis mPFS		
	Numbers	HR (95% CI)	*p* value	Numbers	HR (95% CI)	*p* value
Age > 65 vs ≤65	29 vs 51	1.072 (0.631 to 1.819)	0.7980	20 vs 31	0.364 (0.151 to 0.875)	0.0239
ECOG 0/1 vs 2/3/4	47 vs 7	0.395 (0.169 to 0.925)	0.0325	45 vs 6	0.363 (0.141 to 0.935)	0.0359
EGFRm vs EGFR WT	9 vs 21	0.602 (0.235 to 1.542)	0.2904	—	—	—
Male vs Female	48 vs 32	2.277 (1.297 to 3.998)	0.0042	32 vs 19	2.013 (0.911 to 4.450)	0.0837
Smoking history, yes vs no	60 vs 15	3.020 (1.354 to 6.736)	0.0069	40 vs 11	1.983 (0.696 to 5.656)	0.2003
Resectable, yes vs no	13 vs 54	0.341 (0.144 to 0.804)	0.0140	—	—	—
Adenocarcinoma vs Others	49 vs 30	0.592 (0.348 to 1.005)	0.0523	29 vs 22	0.564 (0.247 to 1.284)	0.1721
Surgery in first‐line, yes vs no	13 vs 67	0.328 (0.140 to 0.769)	0.0103	5 vs 46	0.0 (0.0 to NC)	0.9903
cCRT alone in first‐line, yes vs no	15 vs 65	1.546 (0.819 to 2.921)	0.1790	10 vs 41	0.678 (0.271 to 1.697)	0.4069
cCRT alone in first‐line vs sCRT alone in first‐line	15 vs 9	1.876 (0.730 to 4.821)	0.1911	—	—	—
Trimodality in first‐line, yes vs no	6 vs 74	0.819 (0.296 to 2.269)	0.7015	3 vs 48	652,831 (0.0 to NC)	0.9912

	Stage IIIB					
	Univariate analysis mOS			Multi‐variate analysis mOS		
	Numbers	HR (95% CI)	*p* value	Numbers	HR (95% CI)	*p* value
Age > 65 vs ≤65	29 vs 50	1.278 (0.667 to 2.451)	0.4596	20 vs 31	0.516 (0.195 to 1.365)	0.1826
ECOG 0/1 vs 2/3/4	47 vs 7	0.343 (0.137 to 0.862)	0.0229	45 vs 6	0.282 (0.096 to 0.825)	0.0208
EGFRm vs EGFR WT	9 vs 20	0.738 (0.231 to 2.364)	0.6094	—	—	—
Male vs Female	47 vs 32	2.109 (1.059 to 4.200)	0.0337	32 vs 19	1.504 (0.611 to 3.701)	0.3741
Smoking history, yes vs no	59 vs 15	2.281 (0.881 to 5.907)	0.0893	40 vs 11	0.935 (0.286 to 3.058)	0.9114
Resectable, yes vs no	12 vs 54	0.241 (0.073 to 0.802)	0.0203	—	—	
Adenocarcinoma vs Others	48 vs 30	0.400 (0.211 to 0.759)	0.0050	29 vs 22	0.340 (0.126 to 0.915)	0.0326
Surgery in first‐line, yes vs no	12 vs 67	0.159 (0.038 to 0.669)	0.0121	5 vs 46	0.0 (0.0 to NC)	0.9910
cCRT alone in first‐line, yes vs no	15 vs 64	1.831 (0.890 to 3.765)	0.1002	10 vs 41	0.842 (0.314 to 2.259)	0.7333
cCRT alone in first‐line vs sCRT alone in first‐line	15 vs 9	1.718 (0.585 to 5.048)	0.3253	—	—	—
Trimodality in first‐line, yes vs no	5 vs 74	0.340 (0.047 to 2.476)	0.2866	3 vs 48	1.01E6 (0.0 to NC)	0.9917

*Note* 1: Stage of tumour is per AJCC 7th edition.

*Note* 2: In the univariate analysis, each time one variable is considered, only patients with missing data in that variable were excluded. While, in the multi‐variate analysis, patients with missing data in any of the variables input to the model were excluded. So the numbers for univariate and multi‐variate analyses differ. The variables for multi‐variate analysis are based on the univariate analysis results and also the assumptions for multi‐variate Cox proportional hazards model are valid.

Abbreviations: AJCC, American Joint Committee on Cancer; cCRT, concurrent chemoradiotherapy; CI, confidence interval; EGFR, epidermal growth factor receptor; ECOG, Eastern Cooperative Oncology Group; HR, hazard ratio; mOS, median overall survival; mPFS, median progression‐free survival; NC, non‐calculable; NSCLC, non‐small‐cell lung cancer; sCRT, sequential chemoradiotherapy; TNM, tumour, node, metastasis; WT, wild type. ‐ indicates not available.

In the multi‐variate analyses for stage IIIA, patients with age >65 years and those undergoing surgery as part of initial therapy was associated with significantly longer mPFS (HR, 0.23 [95% CI, 0.08–0.65], *p* = 0.0052 and HR, 0.30 [95% CI, 0.10–0.89], *p* = 0.0291), whereas for stage IIIB, patients with age >65 years, with an ECOG performance status score of 0/1 was associated with significantly longer mPFS (HR, 0.36 [95% CI, 0.15–0.88], *p* = 0.0239 and HR, 0.36 [95% CI, 0.14–0.94], *p* = 0.0359) and with an ECOG performance status score of 0/1 was associated with significantly longer mOS (HR, 0.28 [95% CI, 0.1–0.83], *p* = 0.0208). The histology of adenocarcinoma significantly improved mOS (HR, 0.34 [95% CI, 0.13–0.92], *p* = 0.0326) for stage IIIB but had no impact on mPFS.

## 4 DISCUSSION

This is the first report of real‐world data on management and survival outcomes for patients with stage III NSCLC in the LATAM region. The data collected predates the approval of durvalumab, which at the time of reporting is the only IO drug approved for the treatment of patients with unresectable/inoperable NSCLC.^23^

The overall percentage of patients receiving initial therapy was similar in the KINDLE global study and the LATAM subset. The study results show heterogeneity in treatment patterns with >25 treatment modalities used as initial therapy. In spite of this diversity, about 50% of patients received chemotherapy alone, cCRT or sCRT as the most frequent treatment modality. In the KINDLE global study, cCRT was the most frequent modality followed by chemotherapy alone and sCRT.^21^ About 30% of patients underwent surgery in the LATAM subset, whereas in the KINDLE global study, about 20% of patients underwent surgical resection with a much higher percentage of patients (about 75%) receiving adjuvant therapy compared with about 30% in the LATAM subset. Patients who underwent surgery were mostly those with stage IIIA NSCLC in both KINDLE global cohorts and the LATAM subset. Also, as per the current NCCN guidelines, surgery is not recommended for patients with stage IIIB NSCLC given the extent of the tumour.^12^ Similarly, although chemotherapy alone was the most frequent modality in both stages IIIA and IIIB NSCLC, a greater percentage of patients with stage IIIB NSCLC received chemotherapy alone than those with stage IIIA disease.

Treatment patterns vary in different countries in LATAM. In Peru, patients with stage III disease received CRT and rarely surgery as part of multi‐disciplinary care. This could be attributed to the scarcity of thoracic oncology surgeons in the country.^15^ Results from a retrospective cohort study in Brazil in patients with stage IIIB NSCLC showed that 30.4% of patients received cCRT and 8.7% patients received sCRT as part of initial therapy.^24^ Another study (IIIA 20.9%, IIIB 22.4%) showed that only about one‐fourth of patients (24.6%) received curative surgery^25^ that could be attributed to the unequal distribution of thoracic surgeons in the country.^26^ There is heterogeneity in the treatment pattern within Brazil^27^ where although CRT is largely the SoC for treatment of patients with LA NSCLC, results of the retrospective analysis in elderly patients (>65 years) with unresectable LA disease has shown that about 40% of patients received best supportive care and about 30% of patients each received definitive radiotherapy alone or CRT.^28^ There are gaps in implementing the international and national guidelines given the fragmented healthcare system that impedes optimum care for patients.^29^ There is a lack of uniform access to the healthcare system clubbed with diverse access to lung cancer education for the scientific community in the region.^1^

About two‐thirds of patients relapsed after initial therapy. Of the patients who received any second‐line or third‐line treatment, chemotherapy alone was the preferred treatment in the second‐line, whereas chemotherapy alone or targeted therapy was the preferred treatment in the third‐line treatment. The relapse rate was similar in patients with stage IIIA and IIIB NSCLC, whereas it was about 25% higher in patients with unresectable disease than in those with resectable NSCLC similar to that observed in the KINDLE global cohort.

The mPFS of 14.8 months (95% CI, 12.1–18.6) observed in the LATAM subset is similar to the KINDLE global cohort results. The mOS of 48.6 months (95% CI, 34.7 to NC) is substantially higher than that observed for the global cohort (34.9 months, 95% CI, 32.0–38.0). The mPFS and mOS were higher in stage IIIA NSCLC than in stage IIIB disease. Similar observations were noted for patients with the resectable disease compared with unresectable NSCLC. Surgery as part of initial therapy was an independent predictor for PFS in stage IIIA, whereas an ECOG score of 0/1 was an independent predictor for PFS and OS in stage IIIB. Surprisingly, age >65 years showed improved PFS in both stages; however, this could be attributed to small sample size, selection bias, missing data and patient disease characteristics at study entry.

With the advent of immunotherapy, there has been a paradigm shift in the treatment of NSCLC.^30^, ^31^ Adjuvant osimertinib, a third‐generation EGFR‐TKI, has recently been demonstrated to provide longer disease‐free survival in patients with resected stage I to III EGFR‐mutated NSCLC over placebo.^32^ A study in LATAM showed an overall response rate in EGFR‐mutated patients who received TKI to be at 62.5% (95% CI, 50.0%–75.0%) with an mOS of 16.5 months.^33^ Data from the phase 3 PACIFIC study demonstrated that durvalumab, a human monoclonal antibody, improved PFS and OS, irrespective of the biomarker status when used as consolidation therapy after platinum‐based CRT in patients with stage III NSCLC.^31^, ^34^

Results from the LATAM subset in EGFR‐mutated NSCLC patients (*n* = 175) who received TKIs showed mPFS and mOS of 13 months (95% CI, 11.2–14.8) and 36 months (95% CI, 25.4–46.5), respectively. The survival outcome was higher in patients who received TKIs (as first‐ or second‐line therapy) in the EGFR‐mutated patients compared to the wild type (70.8% versus 29.2%; *p* < 0.001) as well as improved PFS (13.0 versus 3.0 months; *p* < 0.001). OS was significantly different between treatment groups (36.0 versus 14.0 months; *p* < 0.001).^35^

Although the study provides insights into treatment practices for stage III NSCLC in the LATAM region, the retrospective design limits the representativeness of the findings to the post‐IO era. Also, being a real‐world study, the data collection was limited to the availability of existing medical records. Indeed, many data points were missing from being reflective of a real‐world study. With a low sample size, country‐specific analyses for treatment patterns and survival outcomes were not conducted. Hence, future studies with a larger sample size are needed to facilitate country‐level analyses considering the heterogeneity in the treatment patterns for NSCLC in the LATAM region. With the advent of newer therapies, the survival outcome is projected to change. Nevertheless, the data from this study will help in establishing baseline data and planning upcoming real‐world studies in the LATAM region. Considering the role of precision treatment and targeted therapy in the management of NSCLC, the incorporation of molecular tumour boards comprising specialists involved in the treatment decisions is important for recommending genetic testing in this patient population. Implementing guideline‐recommended novel treatment strategies such as targeted therapy and immunotherapy through a multi‐disciplinary approach may homogenise treatment and improve clinical outcomes of patients with stage III NSCLC in the LATAM region.

## 5 CONCLUSIONS

In the LATAM subset of the KINDLE global study in patients with stage III NSCLC, heterogeneity in treatment patterns and corresponding clinical outcomes observed in real‐world settings were reported. The national and international guidelines were not uniformly followed in the region. The relapse rates after initial therapy were high, suggesting a need to streamline treatment selection and sequencing to decrease relapse rates after initial therapy.

## AUTHORS' CONTRIBUTION

Conceptualization: Claudio Marcelo Martin, Adrián Puello‐Guerrero, Luis Alberto Mas‐Lopez. Data curation: Claudio Marcelo Martin, Adrián Puello‐Guerrero, Luis Alberto Mas‐Lopez, Saul Campos‐Gómez, Francisco J. Orlando‐Orlandi. Investigation: Claudio Marcelo Martin, Adrián Puello‐Guerrero, Luis Alberto Mas‐Lopez, Saul Campos‐Gómez, Francisco J. Orlando‐Orlandi. Methodology: Claudio Marcelo Martin, Adrián Puello‐Guerrero, Luis Alberto Mas‐Lopez, Saul Campos‐Gómez, Francisco J. Orlando‐Orlandi, Luis Fernando Tejado Gallegos, Reto Huggenberger. Validation: Luis Fernando Tejado Gallegos, Reto Huggenberger. Visualisation, Writing, Review and editing: Claudio Marcelo Martin, Adrián Puello‐Guerrero, Luis Alberto Mas‐Lopez, Saul Campos‐Gómez, Francisco J. Orlando‐Orlandi, Luis Fernando Tejado Gallegos, Reto Huggenberger.

## CONFLICT OF INTEREST

None.

## CLINICAL REGISTRATION NUMBER

NCT03725475.

## DATA PRESENTED AT

KINDLE (global data): Jazieh AR, Onal HC, Tan DSW, et al. Real‐world treatment patterns and clinical outcomes in patients with stage III non‐small cell lung cancer: Results of KINDLE, a multi‐country observational study. *J Thorac Oncol*. 2021;0(0). doi: http://doi.org/10.1016/j.jtho.2021.05.003. ASCO Conference 2020 (Jazieh AR, Onal HC, Tan DSW, et al. Contemporary management and associated outcomes of 3151 patients with stage III non‐small‐cell lung cancer (NSCLC) in a real‐world setting: Results of KINDLE, a multi‐country observational study. *J Clin Oncol*. 2020:38 (15_suppl); 9043–9043 doi: 10.1200/JCO.2020.38.15_suppl.9043).

## ETHICS STATEMENT

Institutional Review Boards/Independent Ethics Committees from all the participating centres approved the study protocol (clinicaltrials.gov, NCT03725475) before the initiation of the study. Patients or next of kin/legal representatives (in case of a deceased patient) were required to provide written informed consent prior to the data collection.

## Supporting information


Data S1

## Data Availability

The data that support the findings of this study are available from the corresponding author upon request.
