# Supplementary material for: Real‐world KINDLE‐Latin America subset data on treatment patterns and clinical outcomes in patients with stage III non‐small‐cell lung cancer
Source: Cancer Med. 2022 Jul 4;12(2):1247–59. doi: 10.1002/cam4.4990 (PMC9883579; doi:10.1002/cam4.4990)
Supplement: Supplementary file 1 — Data S1 [file CAM4-12-1247-s001.docx]

# **Supplementary Material**

**Figure S1. KINDLE Study - Participating Countries in LATAM (N=231)**

Names of participating countries and the number of patients (indicated in brackets, n) that were recruited by each country.

**Table S1. Treatment Patterns in Stage III NSCLC in KINDLE LATAM**

Treatment modalities by line of therapy are indicated. Second-line or third-line therapies are shown regardless of what kind of first-line or second-line therapy, respectively, the patient received.

|  | **N=231** | | |
| --- | --- | --- | --- |
| **Treatment Modalities, n (%)** | **First-line n=202** | **Second-line n=65** | **Third‑line n=18** |
| Surgery alone | 13 (6.4%) | 2 (3.1%) | 1 (5.6%) |
| Surgery+cCRT | 1 (0.5%) | 0 | 0 |
| Surgery+sCRT | 5 (2.5%) | 0 | 0 |
| Surgery+Chemotherapy | 12 (5.9%) | 0 | 0 |
| Surgery+RT | 1 (0.5%) | 1 (1.5%) | 0 |
| cCRT+Surgery | 8 (4.0%) | 0 | 0 |
| Chemotherapy+Surgery | 2 (1.0%) | 0 | 0 |
| Other Surgery^†^ | 16 (7.9%) | 1 (1.5%) | 0 |
| cCRT | 35 (17.3%) | 3 (4.6%) | 0 |
| cCRT+Chemotherapy | 6 (3.0%) | 0 | 0 |
| cCRT+RT | 0 | 0 | 0 |
| cCRT+Immunotherapy | 3 (1.5%) | 0 | 0 |
| cCRT+Targeted Therapy | 1 (0.5%) | 0 | 0 |
| Other cCRT^‡^ | 0 | 0 | 0 |
| sCRT | 21 (10.4%) | 4 (6.2%) | 1 (5.6%) |
| sCRT+Chemotherapy | 0 | 1 (1.5%) | 0 |
| sCRT+RT | 1 (0.5%) | 1 (1.5%) | 0 |
| sCRT+Immunotherapy | 0 | 1 (1.5%) | 0 |
| sCRT+Targeted Therapy | 2 (1.0%) | 0 | 0 |
| Other sCRT^§^ | 0 | 0 | 0 |
| Chemotherapy alone | 50 (24.8%) | 23 (35.4%) | 4 (22.2%) |
| Chemotherapy+Immunotherapy | 0 | 3 (4.6%) | 2 (11.1%) |
| Chemotherapy+Targeted Therapy | 4 (2.0%) | 2 (3.1%) | 1 (5.6%) |
| Chemotherapy+Targeted Therapy+Immunotherapy | 0 | 1 (1.5%) | 0 |
| RT alone | 13 (6.4%) | 5 (7.7%) | 2 (11.1%) |
| RT+Immunotherapy | 1 (0.5%) | 1 (1.5%) | 1 (5.6%) |
| RT+Targeted Therapy | 1 (0.5%) | 1 (1.5%) | 0 |
| RT+Targeted Therapy+Immunotherapy | 0 | 0 | 0 |
| Immunotherapy | 1 (0.5%) | 6 (9.2%) | 2 (11.1%) |
| Targeted Therapy | 5 (2.5%) | 9 (13.8%) | 4 (22.2%) |

cCRT = concurrent chemoradiotherapy; LATAM = Latin America; NSCLC = non-small‑cell lung cancer; RT = radiotherapy; sCRT = sequential chemoradiotherapy

^†^Other Surgery: other therapies used in combination with surgery like cCRT+ Surgery + Chemotherapy, cCRT +Surgery + Radiotherapty, cCRT +Surgery + Chemotherapy + TKI, cCRT +Surgery + sCRT, cCRT + Surgery + Targeted therapy

^‡^Other cCRT: other therapies (excluding surgery) used in combination with cCRT like cCRT+Chemotherapy+cCRT, cCRT+Chemotherapy+Immunotherapy, cCRT+Chemotherapy+Radiotherapy, cCRT+Chemotherapy+Targeted Therapy, cCRT+Immunotherapy+cCRT, cCRT+Radiotherapy+Chemotherapy, cCRT+Radiotherapy+Targeted Therapy, cCRT+sCRT

cCRT+sCRT+Immunotherapy

^§^Other sCRT: other therapies (excluding surgery) used in combination with cCRT like sCRT+Chemotherapy+TKI, sCRT+Immunotherapy+SCRT, sCRT+Radiotherapy+Chemotherapy, sCRT+Targeted Therapy+Chemotherapy, sCRT+Targeted Therapy + Radiotherapy + Immunotherapy

.

**Table S2. Treatment Patterns as per Stage IIIA and IIIB NSCLC (7^th^ Edition AJCC) in KINDLE LATAM**

Treatment modalities by stage (IIIA vs IIIB) and line of therapy are indicated. Second-line or third-line therapies are shown regardless of what kind of first-line or second-line therapy, respectively, the patient received.

|  | **Stage lllA (N=103)** | | | **Stage lllB (N=90)** | | |
| --- | --- | --- | --- | --- | --- | --- |
| **Treatment Modalities, n (%)** | **First‑line (n=89)** | **Second‑line (n=32)** | **Third-line (n=12)** | **First-line (n=79)** | **Second-line (n=26)** | **Third-line (n=6)** |
| Surgery alone | 11 (12.4%) | 1 (3.1%) | 1 (8.3%) | 0 | 1 (3.8%) | 0 |
| Surgery+cCRT | 0 | 0 | 0 | 0 | 0 | 0 |
| Surgery+sCRT | 1 (1.1%) | 0 | 0 | 3 (3.8%) | 0 | 0 |
| Surgery+Chemotherapy | 8 (9.0%) | 0 | 0 | 1 (1.3%) | 0 | 0 |
| Surgery+RT | 0 | 1 (3.1%) | 0 | 0 | 0 | 0 |
| cCRT+Surgery | 5 (5.6%) | 0 | 0 | 3 (3.8%) | 0 | 0 |
| Chemotherapy+Surgery | 2 (2.2%) | 0 | 0 | 0 | 0 | 0 |
| Other Surgery^†^ | 7 (7.9%) | 0 | 0 | 6 (7.6%) | 1 (3.8%) | 0 |
| cCRT | 13 (14.6%) | 0 | 0 | 15 (19.0%) | 2 (7.7%) | 0 |
| cCRT+Chemotherapy | 3 (3.4%) | 0 | 0 | 2 (2.5%) | 0 | 0 |
| cCRT+RT | 0 | 0 | 0 | 0 | 0 | 0 |
| CRT+Immunotherapy | 0 | 0 | 0 | 2 (2.5%) | 0 | 0 |
| cCRT+Targeted Therapy | 1 (1.1%) | 0 | 0 | 0 | 0 | 0 |
| Other cCRT^‡^ | 0 | 0 | 0 | 0 | 0 | 0 |
| sCRT | 9 (10.1%) | 3 (9.4%) | 0 | 9 (11.4%) | 1 (3.8%) | 1 (16.7%) |
| sCRT+Chemotherapy | 0 | 1 (3.1%) | 0 | 0 | 0 | 0 |
| sCRT+RT | 0 | 1 (3.1%) | 0 | 1 (1.3%) | 0 | 0 |
| sCRT+Immunotherapy | 0 | 1 (3.1%) | 0 | 0 | 0 | 0 |
| sCRT+Targeted Therapy | 0 | 0 | 0 | 2 (2.5%) | 0 | 0 |
| Other sCRT^§^ | 0 | 0 | 0 | 0 | 0 | 0 |
| Chemotherapy alone | 17 (19.1%) | 9 (28.1%) | 2 (16.7%) | 26 (32.9%) | 13 (50.0%) | 2 (33.3%) |
| Chemotherapy+Immunotherapy | 0 | 2 (6.3%) | 1 (8.3%) | 0 | 0 | 1 (16.7%) |
| Chemotherapy+Targeted Therapy | 2 (2.2%) | 2 (6.3%) | 1 (8.3%) | 2 (2.5%) | 0 | 0 |
| Chemotherapy+Targeted Therapy+Immunotherapy | 0 | 0 | 0 | 0 | 1 (3.8%) | 0 |
| RT alone | 7 (7.9%) | 1 (3.1%) | 1 (8.3%) | 4 (5.1%) | 3 (11.5%) | 1 (16.7%) |
| RT+Immunotherapy | 0 | 0 | 1 (8.3%) | 1 (1.3%) | 0 | 0 |
| RT+Targeted Therapy | 0 | 0 | 0 | 0 | 1 (3.8%) | 0 |
| RT+Targeted Therapy+Immunotherapy | 0 | 0 | 0 | 0 | 0 | 0 |
| Immunotherapy | 1 (1.1%) | 4 (12.5%) | 2 (16.7%) | 0 | 2 (7.7%) | 0 |
| Targeted Therapy | 2 (2.2%) | 6 (18.8%) | 3 (25.0%) | 2 (2.5%) | 1 (3.8%) | 1 (16.7%) |

AJCC = American Joint Committee on Cancer; cCRT = concurrent chemoradiotherapy; NSCLC = non‑small-cell lung cancer; RT = radiotherapy; sCRT = sequential chemoradiotherapy.

^†^Other Surgery: other therapies used in combination with surgery like cCRT+ Surgery + Chemotherapy, cCRT +Surgery + Radiotherapty, cCRT +Surgery + Chemotherapy + TKI, cCRT +Surgery + sCRT, cCRT + Surgery + Targeted therapy

^‡^Other cCRT: other therapies (excluding surgery) used in combination with cCRT like cCRT+Chemotherapy+cCRT, cCRT+Chemotherapy+Immunotherapy, cCRT+Chemotherapy+Radiotherapy, cCRT+Chemotherapy+Targeted Therapy, cCRT+Immunotherapy+cCRT, cCRT+Radiotherapy+Chemotherapy, cCRT+Radiotherapy+Targeted Therapy, cCRT+sCRT

cCRT+sCRT+Immunotherapy

^§^Other sCRT: other therapies (excluding surgery) used in combination with cCRT like sCRT+Chemotherapy+TKI, sCRT+Immunotherapy+SCRT, sCRT+Radiotherapy+Chemotherapy, sCRT+Targeted Therapy+Chemotherapy, sCRT+Targeted Therapy + Radiotherapy + Immunotherapy

**Table S3. Survival Outcomes per Initial Therapy and Stage IIIA and IIIB in KINDLE LATAM**

|  | **Stage IIIA (N=103)** | | **Stage IIIB (N=90)** | |
| --- | --- | --- | --- | --- |
| **First-line treatment** | **mPFS Months (95% CI)** | **mOS Months (95% CI)** | **mPFS Months (95% CI)** | **mOS Months (95% CI)** |
| Surgery+Chemotherapy | NC (15.21 to NC) | NC (23.75 to NC) | 14.1 (NC to NC) | 34.7 (NC to NC) |
| cCRT | 21.2 (2.73 to NC) | 48.0 (5.72 to NC) | 7.0 (1.84 to 11.04) | 7.9 (2.04 to NC) |
| sCRT | 18.6 (0.66 to 29.47) | 70.9 (0.66 to 70.90) | 17.5 (6.24 to NC) | 20.7 (6.83 to NC) |
| Chemotherapy alone | 9.0 (3.22 to 19.06) | 25.9 (9.46 to 53.22) | 7.3 (3.22 to 9.49) | 18.3 (12.98 to NC) |
| RT alone | 13.4 (0.20 to 17.94) | NC (0.20 to NC) | 4.6 (0.16 to 9.49) | 4.6 (0.43 to 11.14) |
| Targeted Therapy | 20.3 (8.31 to 32.30) | NC (41.76 to NC) | 9.3 (8.02 to 10.64) | NC (8.02 to NC) |

cCRT = concurrent chemoraditherapy; CI = confidence interval; mOS = median overall survival; mPFS = median progression-free survival; NC = not calculable; sCRT = sequential chemoraditherapy.

**Table S4. Survival Outcomes per Initial Therapy and Resectability in KINDLE LATAM**

|  | **Patient Number** | | **mPFS Months (95%CI)** | | **Patient Number** | | **mOS Months (95%CI)** | |
| --- | --- | --- | --- | --- | --- | --- | --- | --- |
| **First-line treatment** | **Resectable** | **Unresectable** | **Resectable** | **Unresectable** | **Resectable** | **Unresectable** | **Resectable** | **Unresectable** |
| Surgery alone | 12 | 1 | NC (11.86 to NC) | 1.7 (NC to NC) | 12 | 1 | NC (NC to NC) | 1.7 (NC to NC) |
| Surgery+cCRT | 1 | 0 | NC (NC to NC) | - | 1 | 0 | NC (NC to NC) | - |
| Surgery+sCRT | 4 | 1 | NC (10.55 to NC) | 8.4 (NC to NC) | 3 | 1 | NC (NC to NC) | NC (NC to NC) |
| Surgery+Chemotherapy | 11 | 1 | 33.8 (15.21 to NC) | NC (NC to NC) | 11 | 1 | NC (32.07 to NC) | NC (NC to NC) |
| Surgery+RT | 0 | 1 | - | 7.0 (NC to NC) | 0 | 1 | - | NC (NC to NC) |
| cCRT+Surgery | 7 | 0 | 14.8 (8.41 to NC) | - | 7 | 0 | 28.5 (13.83 to NC) | - |
| Chemotherapy+Surgery | 1 | 1 | NC (NC to NC) | 12.5 (NC to NC) | 1 | 1 | NC (NC to NC) | NC (NC to NC) |
| Other Surgery^†^ | 14 | 2 | 37.4 (12.25 to NC) | 29.0 (6.31 to 51.61) | 14 | 2 | NC (NC to NC) | 52.5 (NC to NC) |
| cCRT | 0 | 29 | - | 14.7 (5.72 to 33.08) | 0 | 29 | - | 36.5 (7.85 to NC) |
| cCRT+Chemotherapy | 0 | 4 | - | 17.1 (6.05 to 17.15) | 0 | 4 | - | 52.1 (NC to NC) |
| cCRT+ Immunotherapy | 0 | 3 | - | NC (5.39 to NC) | 0 | 3 | - | NC (NC to NC) |
| cCRT+Targeted Therapy | 0 | 1 | - | 27.6 (NC to NC) | 0 | 1 | - | NC (NC to NC) |
| sCRT | 3 | 14 | 23.3 (9.46 to NC) | 15.0 (8.38 to 20.70) | 3 | 14 | NC (NC to NC) | 20.7 (8.38 to NC) |
| sCRT+RT | 0 | 1 | - | 23.2 (NC to NC) | 0 | 1 | - | 23.2 (NC to NC) |
| sCRT+Targeted Therapy | 0 | 2 | - | NC (NC to NC) | 0 | 2 | - | NC (NC to NC) |
| Chemotherapy alone | 5 | 37 | 8.1 (1.74 to NC) | 7.4 (4.30 to 11.07) | 5 | 37 | NC (12.98 to NC) | 19.6 (12.16 to 42.28) |
| Chemotherapy+Targeted Therapy | 0 | 2 | - | 19.8 (14.13 to 25.49) | 0 | 2 | - | 42.9 (NC to NC) |
| RT alone | 1 | 8 | 14.7 (NC to NC) | 7.9 (0.16 to 13.44) | 1 | 8 | 14.7 (NC to NC) | 11.1 (0.20 to NC) |
| RT+ Immunotherapy | 0 | 1 | - | 15.1 (NC to NC) | 0 | 1 | - | 23.1 (NC to NC) |
| RT+Targeted Therapy | 0 | 1 | - | NC (NC to NC) | 0 | 1 | - | NC (NC to NC) |
| Immunotherapy | 0 | 1 | - | 7.6 (NC to NC) | 0 | 1 | - | NC (NC to NC) |
| Targeted Therapy | 0 | 4 | - | 20.3 (8.02 to 32.30) | 0 | 4 | - | 41.8 (8.02 to NC) |

cCRT = concurrent chemoradiotherapy; CI = confidence interval; mOS = median overall survival; mPFS = median progression-free survival; NC = not calculable; sCRT = sequential chemoradiotherapy.

^†^Other Surgery: other therapies used in combination with surgery like cCRT+ Surgery + Chemotherapy, cCRT +Surgery + Radiotherapty, cCRT +Surgery + Chemotherapy + TKI, cCRT +Surgery + sCRT, cCRT + Surgery + Targeted therapy

**Table S5. Univariate and Multi‑variate Analyses for Progression-free Survival in KINDLE LATAM**

|  | **Stage III NSCLC** | | | | | |
| --- | --- | --- | --- | --- | --- | --- |
|  | **Univariate Analysis of mPFS** | | | **Multivariate Analysis of mPFS** | | |
|  | **Numbers** | **HR (95% CI)** | **P value** | **Numbers** | **HR (95% CI)** | **P value** |
| Stage IIIA vs IIIB | 89 vs 80 | 0.745 (0.522 to 1.065) | 0.1062 | 40 vs 51 | 1.088 (0.631 to 1.878) | 0.7608 |
| Age >65 vs ≤65 | 99 vs 104 | 0.703 (0.501 to 0.988) | 0.0425 | 37 vs 54 | 0.420 (0.242 to 0.729) | 0.0021 |
| ECOG 0/1 vs 2/3/4 | 108 vs 13 | 0.426 (0.222 to 0.817) | 0.0102 | 81 vs 10 | 0.347 (0.160 to 0.751) | 0.0072 |
| EGFRm vs EGFR WT | 20 vs 49 | 0.610 (0.328 to 1.135) | 0.1187 | - | - | - |
| Male vs Female | 121 vs 82 | 1.461 (1.031 to 2.071) | 0.0330 | 56 vs 35 | 1.456 (0.810 to 2.617) | 0.2090 |
| Smoking history, yes vs no | 154 vs 33 | 1.768 (1.071 to 2.920) | 0.0259 | 72 vs 19 | 1.784 (0.860 to 3.701) | 0.1197 |
| Resectable, yes vs no | 59 vs 116 | 0.392 (0.257 to 0.600) | <.0001 | - | - | - |
| Adenocarcinoma vs Others | 129 vs 71 | 0.656 (0.463 to 0.932) | 0.0185 | 55 vs 36 | 0.764 (0.428 to 1.363) | 0.3624 |
| Surgery in first‑line, yes vs no | 58 vs 145 | 0.407 (0.268 to 0.617) | <.0001 | 21 vs 70 | 0.402 (0.170 to 0.947) | 0.0371 |
| cCRT alone in first‑line, yes vs no | 35 vs 168 | 1.045 (0.667 to 1.637) | 0.8470 | 16 vs 75 | 0.750 (0.370 to 1.517) | 0.4230 |
| cCRT alone in first‑line vs sCRT alone in first line | 35 vs 21 | 1.207 (0.620 to 2.350) | 0.5796 | - | - | - |
| Trimodality in first‑line, yes vs no | 15 vs 188 | 0.657 (0.322 to 1.343) | 0.2495 | 7 vs 84 | 0.524 (0.130 to 2.102) | 0.3616 |

cCRT = concurrent chemoradiotherapy; CI = confidence interval; EGFR = epidermal growth factor receptor; ECOG = Eastern Cooperative Oncology Group; HR = hazard ratio; mPFS = median progression‑free survival; NSCLC = non-small cell lung cancer; sCRT = sequential chemoradiotherapy; WT = wild type.

Note: In the univariate analysis, each time one variable is considered, only patients with missing data in that variable were excluded. While, in the multi-variate analysis, patients with missing data in any of the variables input to the model were excluded. So the numbers for univariate and multi-variate analysis differ. The variables for multi‑variate analysis are based on the univariate analysis results and also the assumptions for multivariate cox proportional hazards model being valid.

**Table S6. Univariate and Multi‑variate Analyses for Overall Survival**

|  | **Stage III NSCLC** | | | | | |
| --- | --- | --- | --- | --- | --- | --- |
|  | **Univariate Analysis of mOS** | | | **Multivariate Analysis of mOS** | | |
|  | **Numbers** | **HR (95% CI)** | **P value** | **Numbers** | **HR (95% CI)** | **P value** |
| Stage IIIA vs IIIB | 89 vs 79 | 0.576 (0.361 to 0.918) | 0.0204 | 40 vs 51 | 0.766 (0.386 to 1.517) | 0.4440 |
| Age >65 vs ≤65 | 99 vs 103 | 0.906 (0.571 to 1.437) | 0.6738 | 37 vs 54 | 0.634 (0.313 to 1.283) | 0.2052 |
| ECOG 0/1 vs 2/3/4 | 108 vs 13 | 0.371 (0.172 to 0.803) | 0.0118 | 81 vs 10 | 0.384 (0.150 to 0.986) | 0.0466 |
| EGFRm vs EGFR WT | 20 vs 48 | 0.501 (0.188 to 1.340) | 0.1686 | - | - | - |
| Male vs Female | 120 vs 82 | 1.732 (1.065 to 2.817) | 0.0269 | 56 vs 35 | 1.495 (0.706 to 3.164) | 0.2931 |
| Smoking history, yes vs no | 153 vs 33 | 1.422 (0.741 to 2.729) | 0.2892 | 72 vs 19 | 0.922 (0.385 to 2.210) | 0.8561 |
| Resectable, yes vs no | 58 vs 116 | 0.242 (0.126 to 0.467) | <.0001 | - | - | - |
| Adenocarcinoma vs Others | 128 vs 71 | 0.436 (0.274 to 0.692) | 0.0004 | 55 vs 36 | 0.537 (0.254 to 1.137) | 0.1045 |
| Surgery in first‑line, yes vs no | 57 vs 145 | 0.268 (0.140 to 0.514) | <.0001 | 21 vs 70 | 0.217 (0.048 to 0.989) | 0.0484 |
| cCRT alone in first‑line, yes vs no | 35 vs 167 | 1.856 (1.091 to 3.160) | 0.0226 | 16 vs 75 | 1.156 (0.519 to 2.575) | 0.7225 |
| cCRT alone in first‑line vs sCRT alone first‑line | 35 vs 21 | 1.810 (0.753 to 4.348) | 0.1845 | - | - | - |
| Trimodality in first‑line, yes vs no | 14 vs 188 | 0.850 (0.343 to 2.107) | 0.7250 | 7 vs 84 | 1.609 (0.206 to 12.597) | 0.6504 |

cCRT = concurrent chemoradiotherapy;CI = confidence interval; EGFR = epidermal growth factor receptor; ECOG = Eastern Cooperative Oncology Group; HR = hazard ratio; mOS = median overall survival; NSCLC = non-small-cell lung cancer; sCRT = sequential chemoradiotherapy; WT = wild type.

Note: In the univariate analysis, each time one variable is considered, only patients with missing data in that variable were excluded. While, in the multi‑variate analysis, patients with missing data in any of the variables input to the model were excluded. So the numbers for univariate and multi‑variate analysis differ. The variables for multi‑variate analysis are based on the univariate analysis results and also the assumptions for multivariate cox proportional hazards model being valid.
